# Osteochondritis Lesions of the Ischiopubic Area in Young Adolescents

**DOI:** 10.1155/2022/3573419

**Published:** 2022-05-30

**Authors:** Nikolaos Laliotis, Chrysanthos Chrysanthou, Panagiotis Konstandinidis, Lambrini Giannakopoulou, Anestis Moumtzouoglou

**Affiliations:** ^1^Orthopaedic Department, Inter Balkan Medical Center, Asklipiou 10 Pilea 57001 Thessaloniki, Greece; ^2^Radiology Department, Inter Balkan Medical Center, Thessaloniki, Greece

## Abstract

Osteochondritis of the ischiopubic area is a rare disease of children that presents with hip pain and limping. Careful examination and appropriate investigations are essential to establish a definite diagnosis. We report a case series of four children, ages 10–14-year-old, with osteochondritis of the ischiopubic area. Plain X-ray examination showed an area of diffuse irregular calcification of the ischium in two of the children, while in the other two there was an asymmetrical enlargement of the ischiopubic synchondrosis. MRI investigation was the most helpful examination. Bone edema was found in all four children. A calcified mass separated from the host ischium was found in the first two children. The cortex was normal, without irregular destruction. Bone edema of both the ischium and pubic alongside the synchondrosis was found in the following two children, with intact cortices and asymmetrical enlargement. Osteochondritis lesions of the ischium and the ischiopubic area have radiological findings similar to several severe diseases. Bone edema on MRI investigation in children must be properly evaluated. Appropriate radiological examination enabled us to confirm the diagnosis of the osteochondritis and to avoid unnecessary procedures. We want to draw attention to the rare diagnosis of osteochondritis of the ischiopubic area, and the clinical significance, as a cause of hip pain and limping in children.

## 1. Introduction

The definite diagnosis of adolescents with pelvic pain, usually accompanied by a limp, requires a thorough examination. The differential diagnoses include slipped capital femoral epiphysis (SCFE), infection, benign and malignant lesions, and autoimmune disorders. Among the rare diagnoses are osteochondritis lesions. Osteochondritis of the ischiopubic area refers in enlargement and abnormal calcification of the pubic, ischium and the ischipubic synchondrosis. It can be found either as calcified apophysitis due to overuse lesion either as an atypical painful enlargement of the ischiopubic synchondrosis, as described from Van Neck-Odelberg. Plain X-rays may have low specificity and confusing findings. Further radiological examination is required to confirm the diagnosis [1–7].

We present a case series of four children, with osteochondritis lesions in the ischiopubic area, diagnosed in our institution. Our paper combines the results of clinical and radiological investigations that are usually nonspecific and often confusing, providing elements for an accurate diagnosis. Invasive procedures are unnecessary when a diagnosis of osteochondritis lesions (either traumatic or idiopathic) is established.

## 2. Case Series

### 2.1. Patients

During the last 5 years, we identified four children referred in our institution for hip pain and limping, who were diagnosed with osteochondritis of the ischiopubic area. Their ages ranged from 10 to 14 years. Their data regarding the clinical and radiographic examinations were obtained retrospectively from the records Tables 1.

One adolescent participating in high-level football sports presented with a limp and pain in the right hip region that had lasted for 3weeks. He was unable to recall any severe injuries while playing. He was unable to hop on the affected side. Clinical examination showed that the hip movements were unaffected. Palpation revealed localized pain of the right ischium.

**Figure 1:**
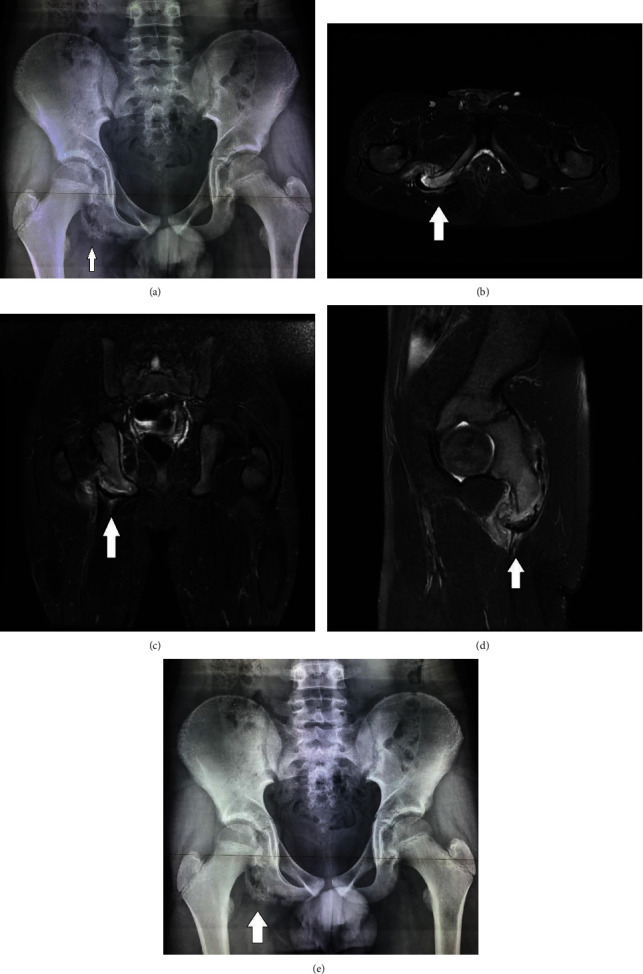
(a) On the right ischium, there is calcification adjacent to the parental bone that has normal structure. (b–d) STIR sequence in transverse (b), coronal (c), and sagittal (d) plane of the pelvis in an adolescent with right hip pain. An asymmetric enlargement of the right ischial tuberosity is observed, with a semilunar osteocartilaginous mass separated from the body of the ischial tuberosity. A fluid field cavity is developed between the parental bone and the detached fragment, whereas concomitant edema of the surrounding muscles due to blood products is also observed. The conjoined hamstring tendon remains attached to the apophysis. (e) There is partial union of the calcified osteochondral bone to the ischium.

The results of initial X-ray examinations of the anterior-posterior (AP) and frog lateral views excluded SCFE. The periphery of the right ischium showed an area of diffuse irregular calcification with smooth boundaries and a normal ischial structure (Figure 1(a)).

Magnetic resonance imaging (MRI) revealed a chondral calcified mass separated by fluid from the host ischium bone. The ischium had a normal structure, without signs of elevated periosteum. The surrounding muscles were edematous and the hamstring tendon was attached to the osteochondral mass. A diagnosis of traumatic avulsion of the ischium resulting from repeated small injuries was established (Figures 1(b)–1(d)).

After 1 month of bed rest and use of crutches for partial weight-bearing (PWB), the patient reported relief of his discomfort. X-rays performed 3 months later showed union of the osteochondral mass to the ischium. The patient returned to his sports activities (Figure 1(e)).

The next two male adolescents presented with pain in the pelvis along with a limp.

Their symptoms had lasted for 3 months and included mild pain that was exacerbated during sports activities. They were also active in football activities, as is common for their age group. Clinical examination showed normal ranges of hip movements, with pain at the end of internal and external rotations. Both adolescents expressed discomfort upon palpation of the ischium tuberosity on the left side.

**Figure 2:**
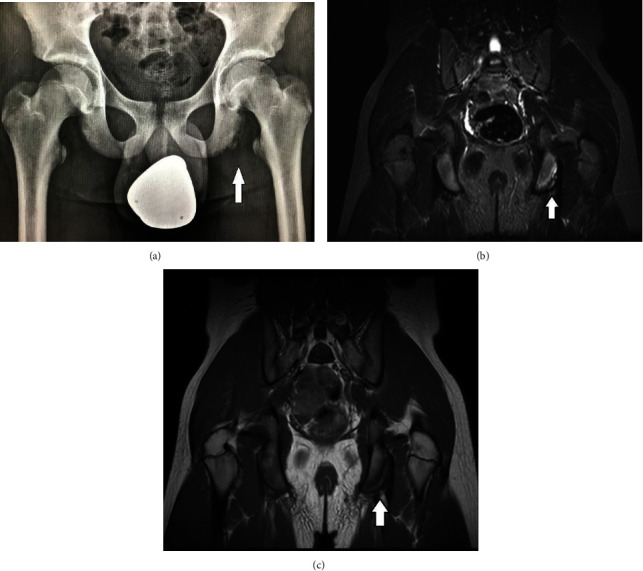
(a) There is an irregular cortex of the left ischial tuberosity, with a smooth line of calcification, in close proximity to the host bone. STIR (b) and T1W (c) sequences in coronal plane of an adolescent with left gluteal pain demonstrate an avulsion lesion of the left ischial tuberosity, with edema of the ischium and minimal displacement of the osteochondral lesion.

X-ray examination of the first child showed signs of irregular cortex on the inferior part of the left ischium, with a smooth line of calcification (Figure 2(a)).

MRI examination revealed bone marrow edema in the ischium and a minimally displaced lesion. The adjacent muscles showed no edema. These were diagnosed as apophyseal lesions of the ischium (Figures 2(b)–2(c)).

**Figure 3:**
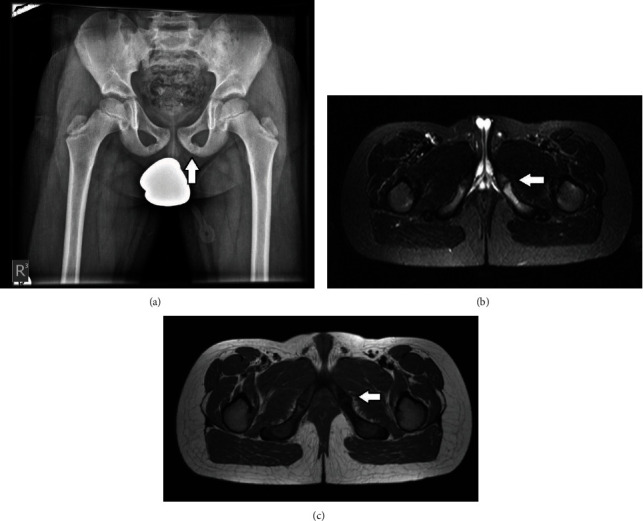
(a) Asymmetric enlargement of the left ischiopubic synchondrosis. (b and c) STIR (b) and T1W (c) sequences in transverse plane in an adolescent with left gluteal pain, demonstrate irregularity of the left ischiopubic synchondrosis, with bone marrow edema involving the bony structures around the synchondrosis. The adjacent soft tissues maintain their normal MRI signal.

X-ray examination of the other boy showed enlargement of the ischiopubic synchondrosis, which was asymmetrically enlarged on the left side (Figure 3(a)). MRI revealed edema on both sides of the synchondroses in the pubic and ischium. These synchondroses appeared wide with irregular lines; however, both the ischium and ilium cortices were normal (Figures 3(b)–3(c)). A Tc99 bone scan showed increased uptake in the area of the synchondrosis on the left side. Thus, the diagnosis of osteochondritis of synchondrosis (Van Neck–Odelberg) was confirmed.

The treatment for both patients was the elimination of the activities and use of crutches for partial weight-bearing, for a month. This treatment resulted in symptom relief and both children returned to their activities after 4–5 months of rest.

The fourth patient was a 10-year-old girl who was initially diagnosed with subacute osteomyelitis of the pelvis. She had experienced pain and discomfort around her left hip that had lasted for 2 months. She was admitted to the pediatric department of a state hospital. MRI revealed edema of the ischiopubic area, which was diagnosed as subacute osteomyelitis, for which treatment with antibiotics was provided.

**Figure 4:**
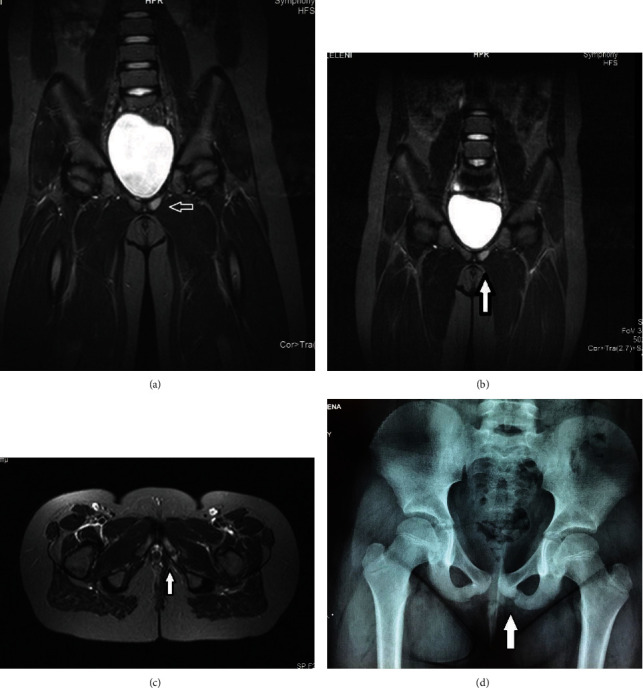
(a–c) STIR sequences in (a and b) coronal and (c) transverse plane demonstrate bone marrow edema of the left ischium, with normal appearance of the cortex and normal appearance of the adjacent muscles. (d) Asymmetric enlargement and diastasis of the left ischiopubic synchondrosis.

Clinical examination revealed a normal active girl with occasional discomfort in activities. A review of the MRI revealed edema of the ischium, with intact periosteum and absence of periosteal reactions. The adjacent muscles were normal (Figures 4(a)–4(c)).

Blood tests showed normal erythrocyte sedimentation rate (ESR), C-reactive protein (CRP), and white blood count. We diagnosed the patient with osteochondritis of the ischiopubic area. Therefore, the patient discontinued the medication and returned to her normal activities within two months. She did not require the use of crutches. X-ray examination confirmed asymmetry of ischiopubic synchondrosis.

## 3. Discussion

Adolescents who present with pain and the hip region accompanied by a limp must be properly evaluated for an accurate diagnosis. It is important to exclude SCFE, which is a commonly missed diagnosis. The potential causes for these symptoms include benign or malignant tumors, infection, autoimmune disorders, and stress fractures [1–3, 5, 6].

Avulsion fractures of the ischium are usually acute injuries, mainly in young athletes, that occur after a sudden contraction of the hamstrings, which present as pain and difficulty in jumping. They are uncommon injuries, mainly observed in adolescents participating in sports activities such as gymnastics, soccer, and dancing. In acute injuries, a history of recent trauma is usually well documented and a curved bony fragment with sharp margins adjacent to the parental bone is typically observed in all imaging modalities, leading to the correct diagnosis [7–9].

Computed tomography (CT) and MRI can be used to the diagnosis, mainly by confirming and evaluating the displacement of the fragment. MRI shows muscle edema; moreover, in cases where plain X-rays cannot detect a bone fragment, this appears as an avulsion lesion on MRI. Depending on the displacement, it can be treated either conservatively or surgically with appropriate stabilization. Surgery is usually indicated for larger fragments, with more than 1.5 cm of avulsion. Painful nonunion may also require surgical stabilization [10–13].

In contrast to typical avulsion ischial tuberosity lesions, our first patient presented with layers of calcification with smooth lining attached to the inferior part of the ischium, which were visible by X-ray. Avulsion injuries in the ischiopubic area in young athletes are usually caused by chronic repetitive trauma. Instead of a distinct detached bony fragment, these injuries present with irregular, ill-defined margins of the parental bone, with concomitant rarefaction and lysis. This type of injury can be mistaken for more aggressive processes, such as infection or Ewing sarcoma, particularly in cases with no clear history of trauma [14].

The radiological image findings showed similarities to osteochondritis of the tibial tuberosity, with layers of calcification. The cortex was intact and there were no areas of moated bone. The periosteal reaction on X-rays must be differentiated from Ewing sarcoma. In sarcoma, MRI shows a destroyed or irregular cortex, with possible extension of the tumor into soft tissues. In our first patient, MRI showed a smooth semilunar ossified mass, with a minimally detached periosteum of the ischium, along with edema of the ischium and fluid between the mass and the host bone. The bone cortex was normal and the hamstring muscles were attached to the bone.

MRI allowed confirmation of the diagnosis of ischium osteochondritis in the second and third adolescent. In their initial radiographs, the second patient had a smooth calcification off the ischium; the third showed irregularity of the ischiopubic synchondrosis, which is a common finding in pelvic X-rays, due to asymmetrical union. Such an asymmetrical union was also reported as a misdiagnosed fracture of the ischiopubic area in a child after a road traffic accident. Proper clinical evaluation and knowledge of asymmetrical union showed normal variation [15]

MRI showed bone edema of the ischium with a small calcified chondral mass attached to the bone in the second patient. The area around the ischium showed no fluid, and the muscles remained attached to the bone. The third patient showed edema in both the ischium and pubic areas, with smooth enlargement and irregularity of the synchondrosis. The cortex and adjacent soft tissues were normal in our fourth patient. Bone edema is the main characteristic of osteochondritis in the growing bone before the completion of apophysis union. It is important to appropriately evaluate the signs of bone edema in growing children. Malignant lesions, including leukemia or Ewing, may also show bone edema but are accompanied by irregular cortex destruction, mass adjacent to the cortex, and asymmetrical destruction. While benign lesions such as osteoid osteoma also show bone edema, the clinical findings differ, and CT scan is the most appropriate method to detect the nidus. On MRI, benign chondral tumors show signs of chondral enlargement. Autoimmune disorders of the pelvis mainly occur in the sacroiliac joint and show joint irregularity and edema on both sides, affecting both the ilium and sacrum.

Irregular enlargement of the ischiopubic joint is usually an area of confusion for accurate diagnosis. Osteochondritis of the synchondrosis, known as “Van Neck–Odelberg,” is characterized by an irregular enlargement of the joint on X-ray [16–18].

Painful enlargement of the synchondrosis must be investigated by MRI or CT to confirm the diagnosis. The characteristic MRI finding is a normal cortex off the bone, edema on both sides, and smooth enlargement of the area, in addition to hyperintense signal in T2 and STIR images and hypointensity in T1 sequences.

Diagnostic procedures, including biopsies, are unnecessary when appropriate investigations are performed. Acute or subacute infections show signs of periosteal reaction on plain radiography, along with signs of localized osteoporosis. MRI of cases of infection shows diffuse edema with periosteal reactions and signs of diffuse edema of the adjacent muscle (peripheral enhancement). Blood tests with elevated ESR and CRP levels are helpful in acute infections but may be normal or slightly elevated in subacute infections. Our fourth patient had edema of the ischium with a normal cortex and no muscle edema. In cases of acute or subacute osteomyelitis, an extended irregular area of edema is expected, with periosteal reaction and periosteal edema [19–22]. In the recent report for Schneider et al., only 2 patients had a diagnostic biopsy, one for possible metastasis and the other to exclude osteomyelitis with histological and microbiological exams [5].

Stress fractures of the ischium are rare. Radiological examinations show periosteal reaction and calcification on both sides of the ischium. In MRI, edema is the main feature, in the presence of a hypointense line perpendicular to the bone line, that is, the fracture line. CT scans further increase the diagnostic ability by showing disruption of the cortex [23].

We managed our patients with bed rest and PWB. While physical therapy (PT) is recommended, we have not provided PT since our patients presented with regular ROM after the resting period. Use of nonsteroid anti-inflammatory medication is helpful for children with severe discomfort [17, 18]. Our adolescents returned in their sport activities after a period of 2-5 months, similar to previously reported cases [5].

Osteochondritis of the ischiopubic area is a rare diagnosis that is established after a complete evaluation of children with pain in the pelvic region and a limp.

## 4. Takeaway Message

Osteochondritis of the ischiopubic area remains a condition that is difficult to be diagnosed. Accurate clinical examination and appropriate radiographic evaluation is essential. Calcification of the area, edema, and enlargement of the synchondrosis must be combined in order to avoid unnecessary procedures. Conservative treatment with rest and PWB is the recommended treatment.

## Figures and Tables

**Table 1:** Patient demographic data, clinical and radiological findings, and treatment.

No.	Sex	Age	Activities	Time to referral	Clinical examination	X-ray	MRI	Treatment	Follow-up
1	M	13	Football	3 weeks	Normal hip movements, localized tenderness	Calcification	Calcified osteochondral mass	PWB with crutches 1 month	Return to sports in 3 months
2	M	14	Sports	3 months	Localized tenderness	Irregular cortex, line of calcification	Avulsion lesion, edema ischium	PWB with crutches 1 month	Return to sports in 4 months
3	M	11	Sports	3 months	Localized tenderness	Asymmetrical enlargement of synchondrosis	Edema of both sides of synchondrosis	PWB with crutches 1 month	Return to sports in 5 months
4	F	10	Dancing	2 months	Normal hip movements	Enlargement and diastasis of synchondrosis	Edema of ischium	No treatment	Return to activities in 1 month

## Data Availability

The data used to support the findings of this study are available from the corresponding author upon request.
